# Antimicrobial resistance of pet-derived bacteria in China, 2000–2020

**DOI:** 10.1128/aac.01657-24

**Published:** 2025-03-26

**Authors:** Yu Song, Qi An, Siyu Chen, Hegen Dai, Shizhen Ma, Congming Wu, Yanli Lyu, Jianzhong Shen, Henrike Krüger-Haker, Stefan Schwarz, Lu Wang, Yang Wang, Zhaofei Xia

**Affiliations:** 1 Department of Clinical Veterinary Medicine, College of Veterinary Medicine, China Agricultural University 630101 , Beijing, China; 2 National Key Laboratory of Veterinary Public Health Safety, College of Veterinary Medicine, China Agricultural University 630101 , Beijing, China; 3 Key Laboratory of Animal Antimicrobial Resistance Surveillance, Ministry of Agriculture and Rural Affairs, College of Veterinary Medicine, China Agricultural University 630101 , Beijing, China; 4 Institute of Microbiology and Epizootics, Freie Universität Berlin 685237, Berlin, Germany; 5 Veterinary Centre for Resistance Research (TZR), Freie Universität Berlin 9166, Berlin, Germany; Houston Methodist Hospital and Weill Cornell Medical College, Houston, Texas, USA

**Keywords:** antimicrobial resistance, China, pet, bacteria, prevalence

## Abstract

With the rapid growth of the pet industry in China, bacterial infectious diseases in pets have increased, highlighting the need to monitor antimicrobial resistance (AMR) in pet-derived bacteria to improve the diagnosis and treatment. Before the establishment of the China Antimicrobial Resistance Surveillance Network for Pets (CARPet) in 2021, a comprehensive analysis of such data in China was lacking. Our review of 38 point-prevalence surveys conducted between 2000 and 2020 revealed increasing trends in AMR among pet-derived *Escherichia coli*, *Klebsiella pneumoniae*, *Staphylococcus* spp., *Enterococcus* spp., and other bacterial pathogens in China. Notable resistance to β-lactams and fluoroquinolones, which are largely used in both pets and livestock animals, was observed. For example, resistance rates for ampicillin and ciprofloxacin in *E. coli* frequently exceeded 50.0%, with up to 41.3% of the isolates producing extended-spectrum β-lactamases. The emergence of carbapenem-resistant *K. pneumoniae* and *E. coli*, carrying *bla*_NDM_ and *bla*_OXA_ genes, highlighted the need for vigilant monitoring. The detection rate of SCC*mec* (Staphylococcal Cassette Chromosome *mec*), a genetic element associated with methicillin resistance, in *Staphylococcus pseudintermedius* isolated from pets in China was found to be over 40.0%. The resistance rate of *E. faecalis* to vancomycin was 2.1% (5/223) in East China, which was higher than the detection rate of human-derived vancomycin-resistant *Enterococcus* (0.1%, 12/11,215). Establishing the national AMR surveillance network CARPet was crucial, focusing on representative cities, diverse clinical samples, and including both commonly used antimicrobial agents in veterinary practice and critically important antimicrobial agents for human medicine, such as carbapenems, tigecycline, and vancomycin.

## INTRODUCTION

Over the past two decades, as living standards have improved, pets have become important members of many families. In China, the number of urban dogs and cats reached 121.55 million in 2023, representing a 4.3% increase compared to 2022. This includes 51.75 million dogs, a slight increase of 1.1% from 2022, and 69.80 million cats, reflecting a 6.8% rise from the previous year (1). The close contact between pets and humans facilitates the transmission of zoonotic bacteria, including antimicrobial-resistant pathogens (2–5). Veterinarians and animal hospital staff are also at risk of exposure and transmission (6–9). Therefore, monitoring and analyzing the antimicrobial resistance (AMR) of pet-derived bacteria is essential for improving the diagnosis and treatment of bacterial infections in pets. Before the establishment of the China Antimicrobial Resistance Surveillance Network for Pets (CARPet) in 2021, there was no comprehensive analysis of AMR in pet-derived bacteria across China. This review of point-prevalence surveys (PPS) from 2000 to 2020 summarizes the trends in AMR among pet-derived bacteria in China, providing a basis for future resistance monitoring both nationally and globally.

## USAGE OF ANTIMICROBIAL AGENTS IN THE CHINESE PET INDUSTRY

The global consumption of antimicrobial agents has risen alongside the growing human population and the increasing demand for animal-derived food products. China has emerged as a leading producer and consumer of antimicrobial agents for both animals and humans (10). According to the 2018 Status Report on Antimicrobial Management and Bacterial Resistance released by the National Health Commission, the top five antimicrobial agents used for treating human inpatients in China in 2017 were fluoroquinolones (13.1%), third-generation cephalosporins (12.9%), cephalosporins/enzyme inhibitors (11.6%), second-generation cephalosporins (9.8%), and penicillins/enzyme inhibitors (7.7%) (11). In addition, the Veterinary Bulletin issued by the Chinese Ministry of Agriculture and Rural Affairs reported that the total amount of antimicrobial agents used in animals in China was 32,776 tons in 2020, with the top three being tetracyclines (30.5%), sulfonamides and synergists (13.1%), and β-lactams/β-lactamase inhibitors (12.6%). When categorized by usage purpose, 28.7% was for growth promotion and 71.3% was for therapeutic use. Among the antimicrobial agents used for growth promotion, chlortetracycline accounted for the largest share (79.0%). Other agents used for this purpose included bacitracin, oxytetracycline, tilmicosin, enramycin, streptomycin, nisin, and avermectin (12).

The substantial increase in the number of pets has led to a corresponding rise in the use of antimicrobial agents for pets. According to the Chinese Pet Anti-Infective Drugs Market Survey Report, the annual sales volume of pet antimicrobial agents in China grew from 4,919,000 units in 2016 to 10,505,000 units in 2021, with projections suggesting it will reach 28,663,000 units by 2027 (13). It is important to note that “units” in this context refers to the measurement of finished medications, vaccines, and vitamins, with the volume or mass of one unit varying depending on the concentration or potency of the substance. While the general metric for active substances is the metric ton, the survey report specifically accounted for finished products rather than raw weight (Table 1). To clarify, the figures in Table 1 refer exclusively to antimicrobial agents and do not include other compounds, such as vitamins and vaccines. A 2016 survey conducted in North China revealed that amoxicillin/clavulanate, enrofloxacin, and ceftriaxone were the most commonly used antimicrobial agents for dogs and cats (14). Similarly in Europe, the combination of amoxicillin/clavulanic acid was the predominant clinical antimicrobial agent for pets. Specifically, the use of third- and fourth-generation cephalosporins in cats was higher (21.0%) than in dogs (1.0%) (15). Moreover, the usage of major antimicrobial agents varied not only between dogs and cats but also depending on the site of infection. In Europe, statistics indicated that cephalosporins and penicillins were commonly used to treat skin diseases in both species. However, due to differences in indications for these two drug classes, their usage proportions in clinical practice varied. For instance, penicillins accounted for 51.0% of usage in cats, as the most common skin conditions were bite wounds and abscesses. In contrast, the predominant skin conditions in dogs were pyoderma and otitis, for the treatment of which both first- and second-generation cephalosporins and penicillins were used, each accounting for approximately 30.0% of usage (15).

**TABLE 1:** Sales volumes and forecast of pet anti-infective drugs in China (2016–2027) (K units) (13)

	East China	South China	North China	Central China	West China	Other regions	Total
2016	1,448	788	1,026	722	352	583	4,919
2017	1,695	928	1,203	856	416	676	5,774
2018	1,948	1,073	1,370	990	483	760	6,624
2019	2,211	1,224	1,545	1,123	547	854	7,504
2020	2,645	1,489	1,862	1,360	627	1,017	9,045
2021	3,046	1,750	2,153	1,596	790	1,170	10,505
2022	3,523	2,088	2,557	1,893	942	1,391	12,394
2023	4,187	2,492	3,034	2,259	1,134	1,637	14,743
2024	4,989	3,004	3,627	2,735	1,371	1,941	17,667
2025	5,846	3,614	4,260	3,254	1,650	2,267	20,909
2026	6,914	4,293	4,978	3,834	1,969	2,626	24,614
2027	7,894	5,100	5,806	4,483	2,331	3,049	28,663

## AMR OF PET-DERIVED BACTERIA IN CHINA

We reviewed PPS reporting AMR rates in pets across China from 2000 to 2020 (Supplementary Text S1), focusing on the two most common pet species: dogs and cats. The review targeted seven major bacterial pathogens: *Escherichia coli*, *Klebsiella pneumoniae*, *Staphylococcus* spp., *Enterococcus* spp., *Salmonella* spp., *Pseudomonas aeruginosa* and *Proteus mirabilis*. We recorded the reported resistance rates and genotypes and also extracted relevant information, such as publication year, the types of antimicrobial agents used for testing, methods used for susceptibility testing, breakpoints used for classification of isolates as susceptible, intermediate, or resistant, sample source locations, number of samples collected, and the host animals involved (Table S1). Samples investigated in each survey included a variety of body sites, such as urinary tract, nasopharynx, abscesses, skin, and ear canal, among others, with each sample collected from a single animal. Finally, 38 articles were included in the Chinese pet AMR survey, encompassing 3,847 isolates collected from Northwest, Northeast, North, Central, Southwest, and South China.

**Fig 1:**
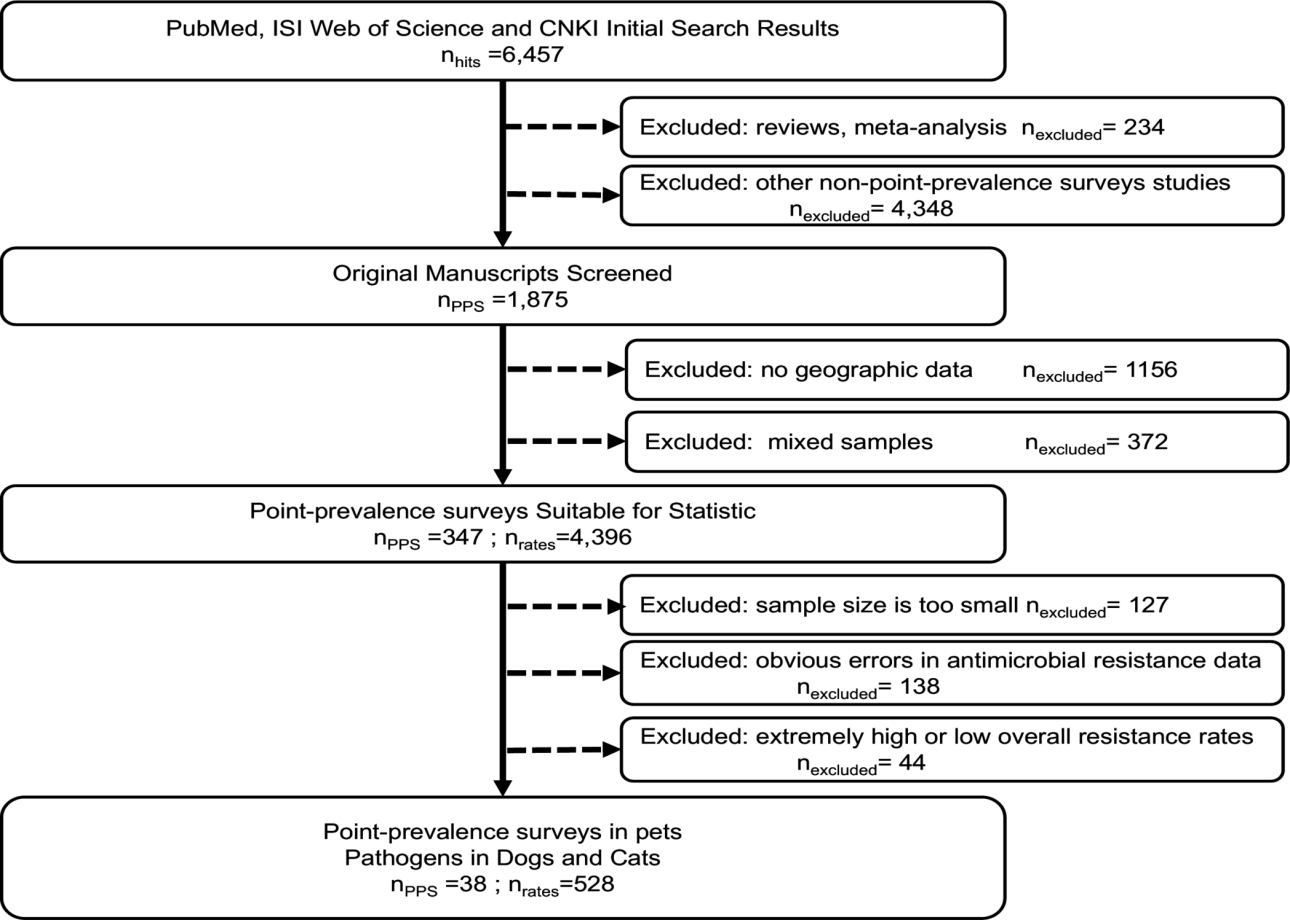
Procedure of literature review showing the number of resistance rates (*n*_*r*ates_) and point-prevalence surveys (*n*_PPS_) identified, along with the exclusion criteria and records used for mapping antimicrobial resistance.

To ensure the comparability of AMR rates across surveys, we extracted drug–bacteria combinations recommended for susceptibility testing by the World Health Organization Advisory Group on Integrated Surveillance of AMR (16). To minimize discrepancies arising from different antimicrobial susceptibility test methods, we standardized our selection to include studies using dilution methods (e.g., broth microdilution) and diffusion methods (e.g., agar disc diffusion). Previous research has demonstrated strong concordance in measuring resistance in animal-derived bacteria using these methods, leading to the extraction of 528 resistance rates across 83 drug–bacteria combinations (Fig. 1). We calculated the weighted arithmetic mean of AMR to summarize trends in resistance across multiple antimicrobial agents and bacterial species (Supplementary Text S2).

### E. coli

**Fig 2:**
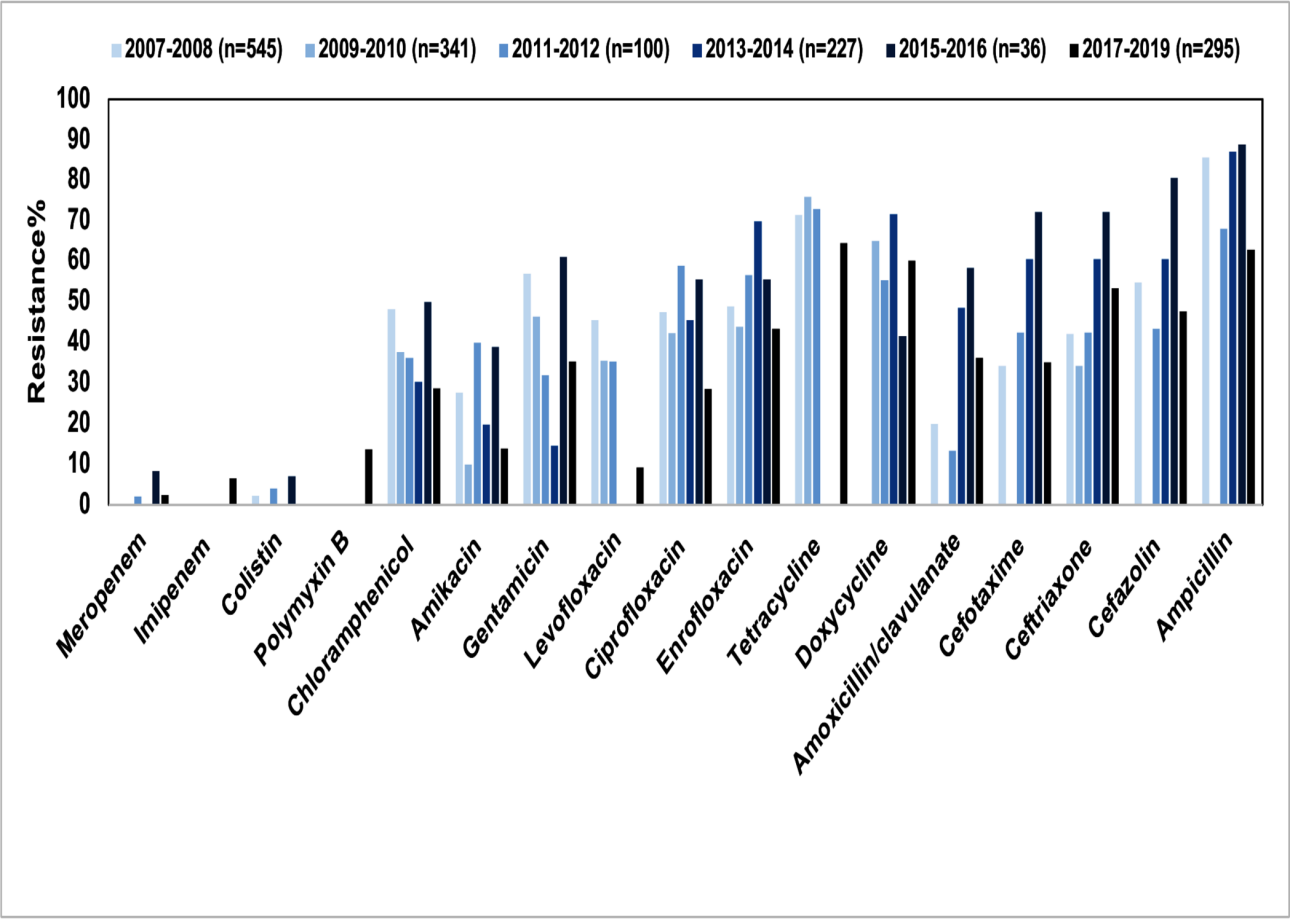
Overall antimicrobial resistance of pet-derived *E. coli* in China, 2007–2019.

**Fig 3:**
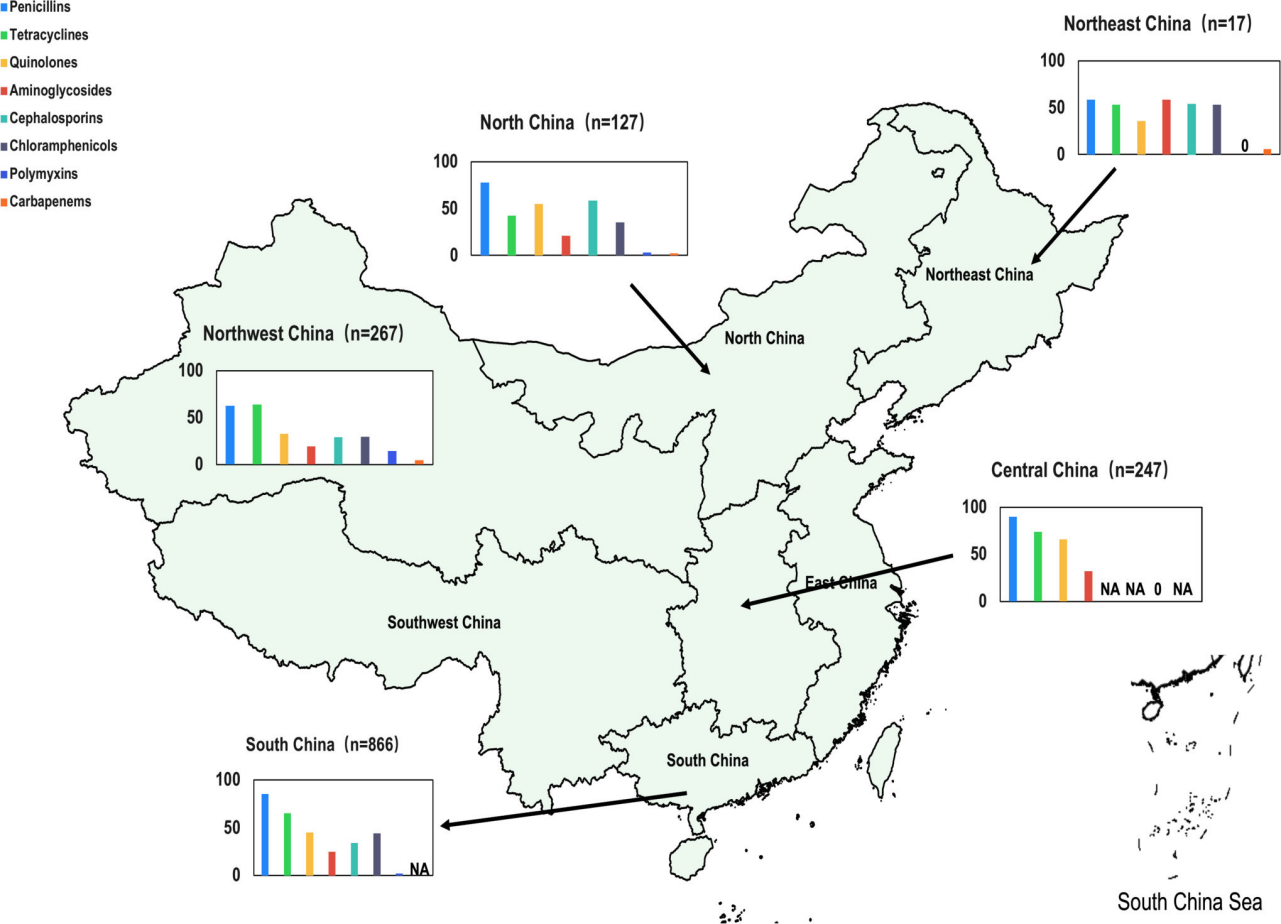
Overall antimicrobial resistance of pet-derived *E. coli* in different regions of China. NA: No resistance rates data available for *E. coli* isolates for this antimicrobial agent in the region; 0: The resistance rate of s of *E. coli* to this antimicrobial agent were 0.0%. The map was sourced from “ChinaAdminDivisonSHP” and downloaded from Zenodo (17).

*E. coli* is an important pathogen responsible for both intestinal and extraintestinal infections in pets. Multiple studies have reported on the AMR of *E. coli* isolates from pets in China. These isolates were mainly collected from the feces and perianal regions of healthy pets, as well as from diarrheic pets and various extraintestinal infection sites, such as urine, nasopharyngeal swabs, eyes, blood, abscesses, skin, and ear canals. The resistance rates of *E. coli* isolates to most antimicrobial agents were generally greater than 50.0%, with variations observed annually. Notably, *E. coli* from healthy pets exhibited lower resistance rates compared to those from diseased pets (Fig. 2 and Supplementary Excel file). In general, *E. coli* most frequently showed resistance to β-lactams (ampicillin 62.9–88.9%, cefazolin 43.3–80.6%, and ceftriaxone 34.3–72.2%), fluoroquinolones (ciprofloxacin 43.0–66.7%, enrofloxacin 43.4–69.9%, and levofloxacin 9.2–45.4%), and tetracyclines (tetracycline 64.4–72.9% and doxycycline 41.7–71.6%), which were extensively used in both pets and food-producing animals. Considering aminoglycosides, another class of commonly used antimicrobial agents, relatively high resistance rates were also observed, with *E. coli* isolates from pets exhibiting generally above 40.0% resistance to gentamicin and around 30.0% resistance to amikacin (Fig. 2). Regional variations in AMR among *E. coli* isolates from pets in China revealed both similarities and distinct differences in resistance levels to antimicrobial agents. In isolates from South and Central China, resistance rates to penicillins (85.6–90.0%) and tetracyclines (65.1–73.9%) were notably high, while isolates from Northeast China exhibited elevated resistance rates to aminoglycosides (58.8%) and cephalosporins (52.9%). Isolates from Northwest China showed a notably high resistance rate to colistin (14.8%), highlighting the need for strengthened monitoring in this region. In contrast, carbapenem resistance rates remained relatively low across the country (2.4–5.9%), indicating manageable risks at present. These findings suggest the critical need for region-specific antimicrobial usage policies and tailored resistance monitoring strategies. Enhanced surveillance of colistin resistance in isolates from Northwest China is especially critical, along with broader nationwide AMR mitigation efforts to protect public health and ensure the safety of veterinary treatments (Fig. 3).

The production of extended-spectrum β-lactamases (ESBLs) by *E. coli* isolated from pets is a significant concern. The production of ESBLs in the isolates was determined with the phenotypic confirmatory test using both cefotaxime and ceftazidime, alone and in combination with clavulanic acid (18). In a study of *E. coli* isolates from pets in South China, 41.3% (99/240) were confirmed to produce ESBLs. Notably, the ESBL-positive rate was significantly higher in isolates from diseased pets (54.5%, 73/134) compared to those from healthy pets (24.5%, 26/106) (19). Similar studies in Northwest China reported ESBL rates of 29.2% (35/120), 24.2% (40/165), and 20.0% (20/100) (20–22). The ESBL rates of *E. coli* varied widely across different hosts and regions. Notably, the rates in pet-derived *E. coli* in China were higher than those reported in the United States (3.0%, 29/944) (23) and Switzerland (7.5%, 8/107) (24) and were comparable to rates in human-derived *E. coli* in China (52.6%, 42,400/80,609) (25).

The most common ESBL genotype in pet *E. coli* in China was *bla*_CTX-M_. The positive rates of *bla*_CTX-M_ among ESBL-producing *E. coli* across various studies ranged from 68.6% to 99.0%, with *bla*_CTX-M-14_, *bla*_CTX-M-55_, and *bla*_CTX-M-15_ being the predominant variants (19–21, 26–28). In addition, other β-lactam resistance genes, including *bla*_SHV-12_, *bla*_OXA-48_, *bla*_TEM-30_, *bla*_CMY-2_, and *bla*_DHA-1_, were also detected in ESBL-producing *E. coli* isolates from pets in China (21). Moreover, *bla*_CTX-M_ commonly co-occurred with other resistance genes in *E. coli* isolated from Chinese pets. For example, in South China, all pet *E. coli* carrying the fosfomycin resistance gene *fosA3* (*n* = 29) also harbored *bla*_CTX-M_ (29). Another study in Northwest China found that all *bla*_CTX-M_-positive *E. coli* isolates were associated with the plasmid-mediated quinolone resistance (PMQR) gene *aac(6′)-Ib-cr* (*n* = 22) (21). The co-existence of ESBL and PMQR genes was also common in *E. coli* from companion and food-producing animals in China (30). Among the fluoroquinolone-resistant *E. coli* isolated from pets, *qnrS*, and *aac(6′)-Ib-cr* were the most frequently detected PMQR genes in China, with their prevalence exceeding 14.0% in most studies (21, 31–33). In addition, studies on resistance to other antimicrobial agents commonly used in animals, such as tetracyclines and aminoglycosides, revealed high carriage rates of *tet*(A) (57.4–62.0%), *aac(6')-lb* (17.0–18.14%), and *ant(3″)-Ia* (12.8–22.12%) in pet-derived *E. coli* from Northwest and Central China (28, 31, 34).

Although *E. coli* isolates from pets in China commonly exhibited resistance to antimicrobials used in veterinary clinics, they were infrequently resistant to antimicrobials currently restricted from being used for animals in China. For instance, in most studies conducted in China, the resistance rates of pet-derived *E. coli* to carbapenems were below 10.0% (Fig. 2). The primary mechanism responsible for carbapenem resistance in pet-derived *E. coli* was the metallo-β-lactamase-encoding gene *bla*_NDM-5_ (26, 35), while the OXA-type β-lactamase-encoding gene *bla*_OXA-48_ was also detected in some reports (21). Although colistin was widely used as a growth promoter in food animals in China before 2017, the pet-derived *E. coli* isolates infrequently exhibited resistance to colistin. However, it is noteworthy that the resistance rate of pet-derived *E. coli* to colistin in China increased annually, rising from 0.0% to 2.0% in 2007 to 7.0% to 18.0% in 2019 (Fig. 2). *E. coli* isolates from pets in Northwest China were more frequently resistant to colistin compared to those from other regions (Fig. 3). It should be noted that carbapenems and colistin are among the few antimicrobial agents available in human medicine for treating multiresistant gram-negative bacteria. Notably, two *E. coli* isolates from pets in China were identified to carry both the carbapenem resistance gene *bla*_NDM-5_ and the colistin resistance gene *mcr-1*. These genes co-existed on a transferable IncX3 plasmid and could be co-transferred (36). These findings suggest that pet-derived *E. coli* isolates not only exhibited high resistance rates to commonly used antimicrobial agents, but might also carry genes that confer resistance to critically important antimicrobial agents, such as carbapenems or colistin. These latter genes potentially undergo horizontal gene transfer and pose a significant threat to the health of both pets and humans.

### K. pneumoniae

**Fig 4:**
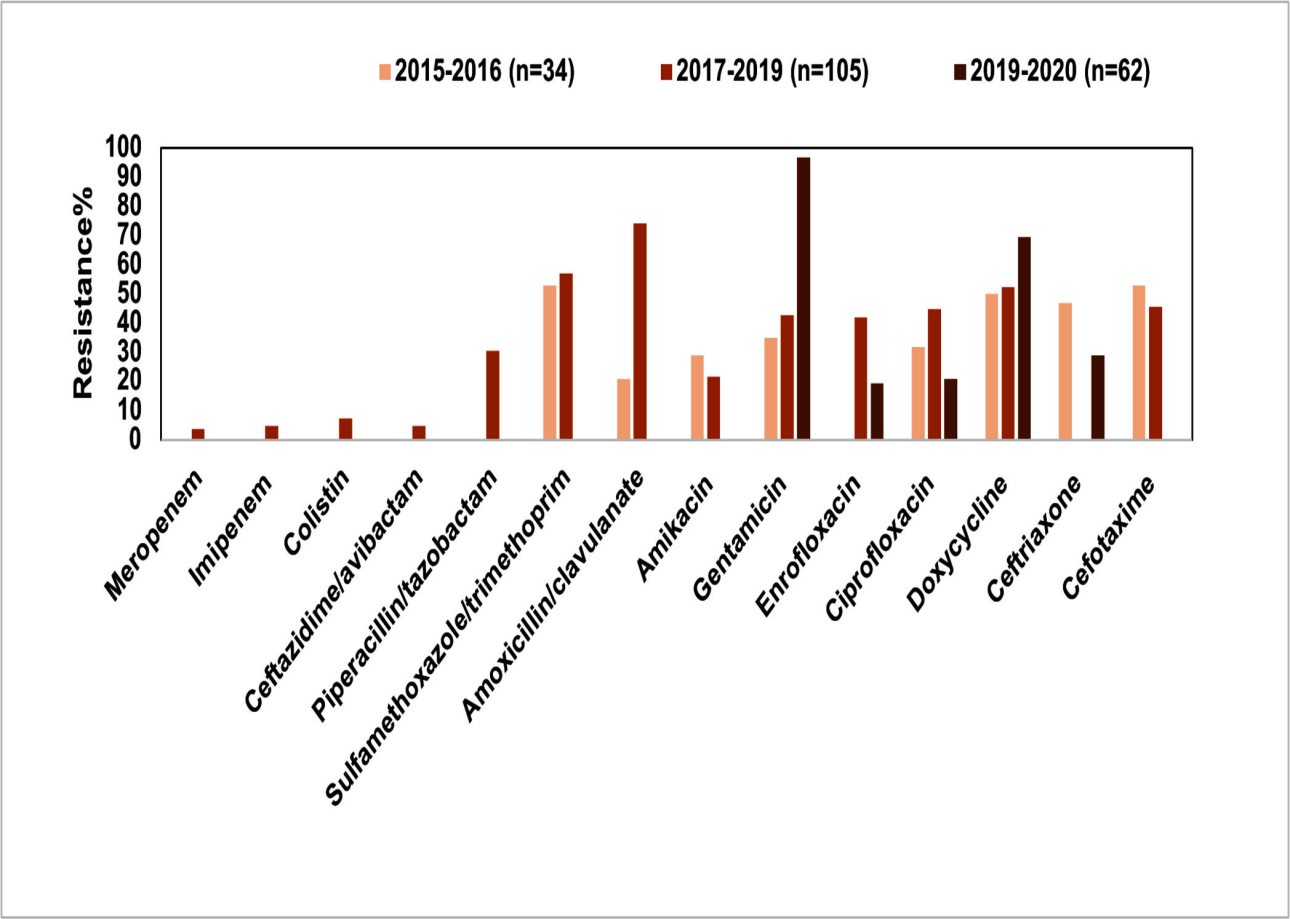
Overall antimicrobial resistance of pet-derived *K. pneumoniae* in China, 2015–2020.

*K. pneumoniae* is an opportunistic pathogen and the second most common species of Enterobacterales, capable of colonizing the skin, upper respiratory tract, and digestive tract of animals (37). In small animal clinics, *K. pneumoniae* is the third most common pathogen responsible for urinary tract infections (UTIs) and bacteremia (38). It can cause varying degrees of infection in young, elderly, and immunocompromised dogs and cats. However, there have been relatively few reports on the AMR of *K. pneumoniae* in pets from North, South, and East China. The isolates studied were mainly collected from urine, nasopharyngeal swabs, abscesses, skin swabs, and ear canal samples. A study conducted in North China provided detailed statistics on the sample sources, with a significant proportion coming from urine (35.2%, *n* = 37), followed by throat swabs (12.4%, *n* = 13), abscesses (9.5%, *n* = 10), and nasal swabs (8.6%, *n* = 9) (39). The resistance rates of *K. pneumoniae* from pets to commonly used antimicrobial agents, such as aminoglycosides, tetracyclines, third-generation cephalosporins, and combination antibiotics, were generally above 30.0% (Fig. 4). Notably, the resistance rates to gentamicin (35.0–96.8%) and doxycycline (50.0–69.4%) have shown a gradual increase over time.

At the molecular level, the diversity of resistance genes in *K. pneumoniae* isolated from pets was considerable. The AMR rates and corresponding resistance genes of *K. pneumoniae* isolated from different regions and sample sites in China exhibited similar patterns. For instance, the aminoglycoside resistance genes *aac(6′)-Ib-cr*, *aac (3)-IId*, *aph (6)-Id*, *aph(3′)-I*, and *aadA* were present in over 30.0% of the isolates, consistent with the aminoglycoside resistance rates. The resistance rates of *K. pneumoniae* to cephalosporins, such as cefotaxime (45.7–53.0%) and ceftriaxone (29.0–47.0%), were also high (Fig. 4), aligning with the high detection rates of the β-lactamase genes *bla*_SHV_, *bla*_TEM_, and *bla*_CTX-M_, which ranged from 41.0% to 100.0% (39–42).

It is noteworthy that few *K. pneumoniae* isolates from pets in China exhibited resistance to carbapenems (39, 43). A 2021 study from North China reported that the resistance rates of *K. pneumoniae* to imipenem and meropenem were 4.8% (5/105) and 3.8% (4/105), respectively. The carbapenem resistance genes *bla*_NDM_ and *bla*_OXA_ were detected in these isolates (39). In addition to carbapenem resistance, resistance to colistin was also observed. In Central China, the resistance rate of *K. pneumoniae* isolates to colistin was 7.6% (8/105), with two *mcr*-positive strains detected (39), while another *mcr*-positive strain was identified in South China (43). A study on colistin-resistant *K. pneumoniae* from pets in Northeast China showed a detection rate of *mcr* genes at 16.9% (201/1,190), with *mcr*-1 being more prevalent in *K. pneumoniae* from pets than other *mcr* variants (44). The detection of carbapenem and colistin resistance phenotypes, along with their associated resistance genes, highlighted the need for increased vigilance regarding the potential spread of these extensively drug-resistant *K. pneumoniae* strains between pets and humans. The emergence of these *K. pneumoniae* strains from pets posed an emerging challenge for the treatment of clinical infections in veterinary practice.

### *Staphylococcus* spp.

**Fig 5:**
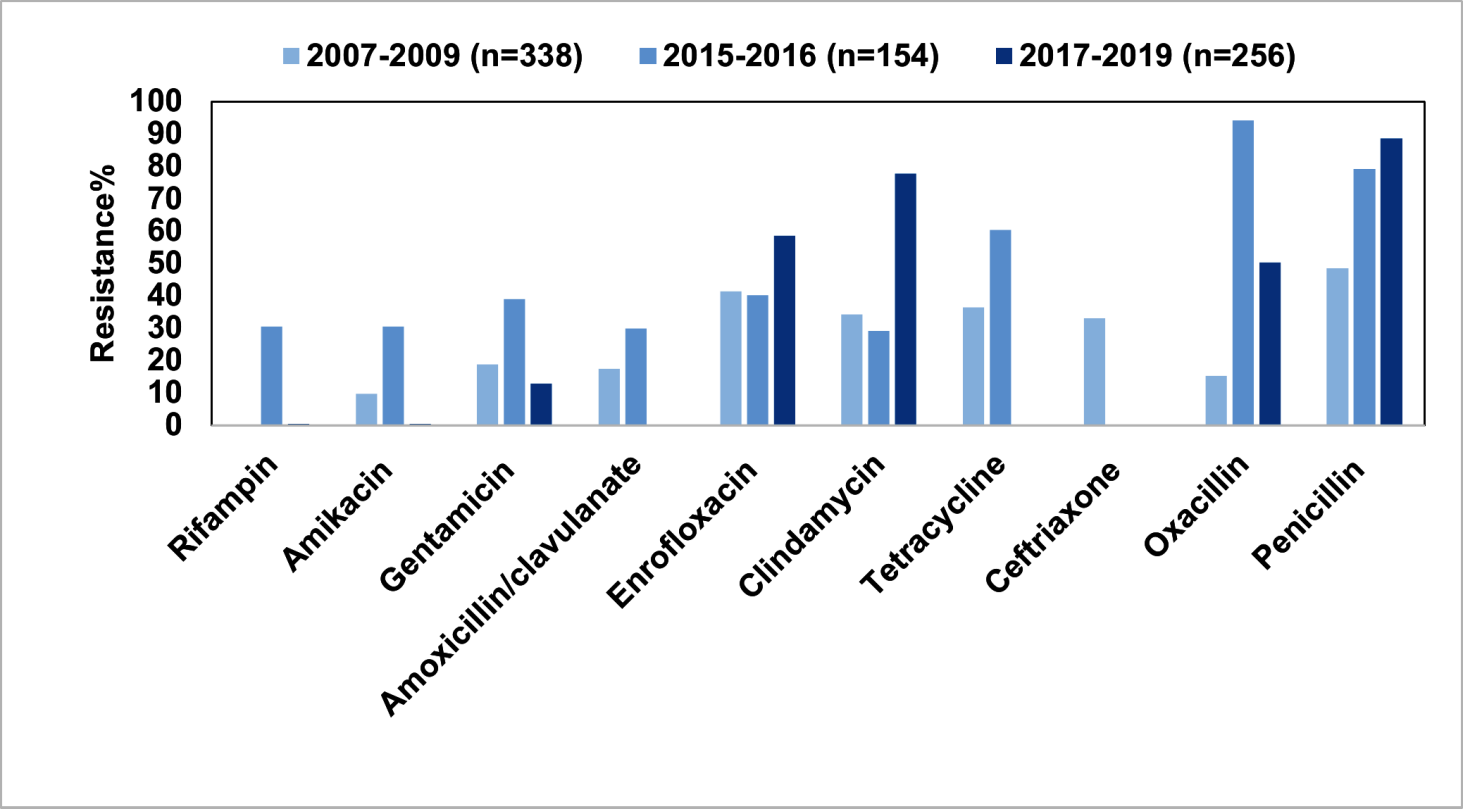
Overall antimicrobial resistance of pet-derived *Staphylococcus* spp. in China, 2007–2019.

*Staphylococcus* spp. are gram-positive cocci widely distributed on the body surface and within cavities connected to the exterior in both humans and animals, with most of them being non-pathogenic. However, *Staphylococcus aureus*, *Staphylococcus epidermidis*, and *Staphylococcus pseudintermedius* are the major *Staphylococcus* spp. responsible for diseases in humans and animals. In China, *Staphylococcus* spp. isolated from diseased pets were primarily collected from the skin (*n* = 223) (45, 46), the external auditory meatus (*n* = 64) (45), and the throat (*n* = 50) (47). Among them, *S. pseudintermedius*—a significant reservoir of AMR genes and common in dogs and cats (48)—had the highest isolation rate. The resistance rates of *Staphylococcus* spp. from pets to fluoroquinolones, tetracycline, and penicillin were relatively high (>50.0%) and have gradually increased over time (Fig. 5).

**Fig 6:**
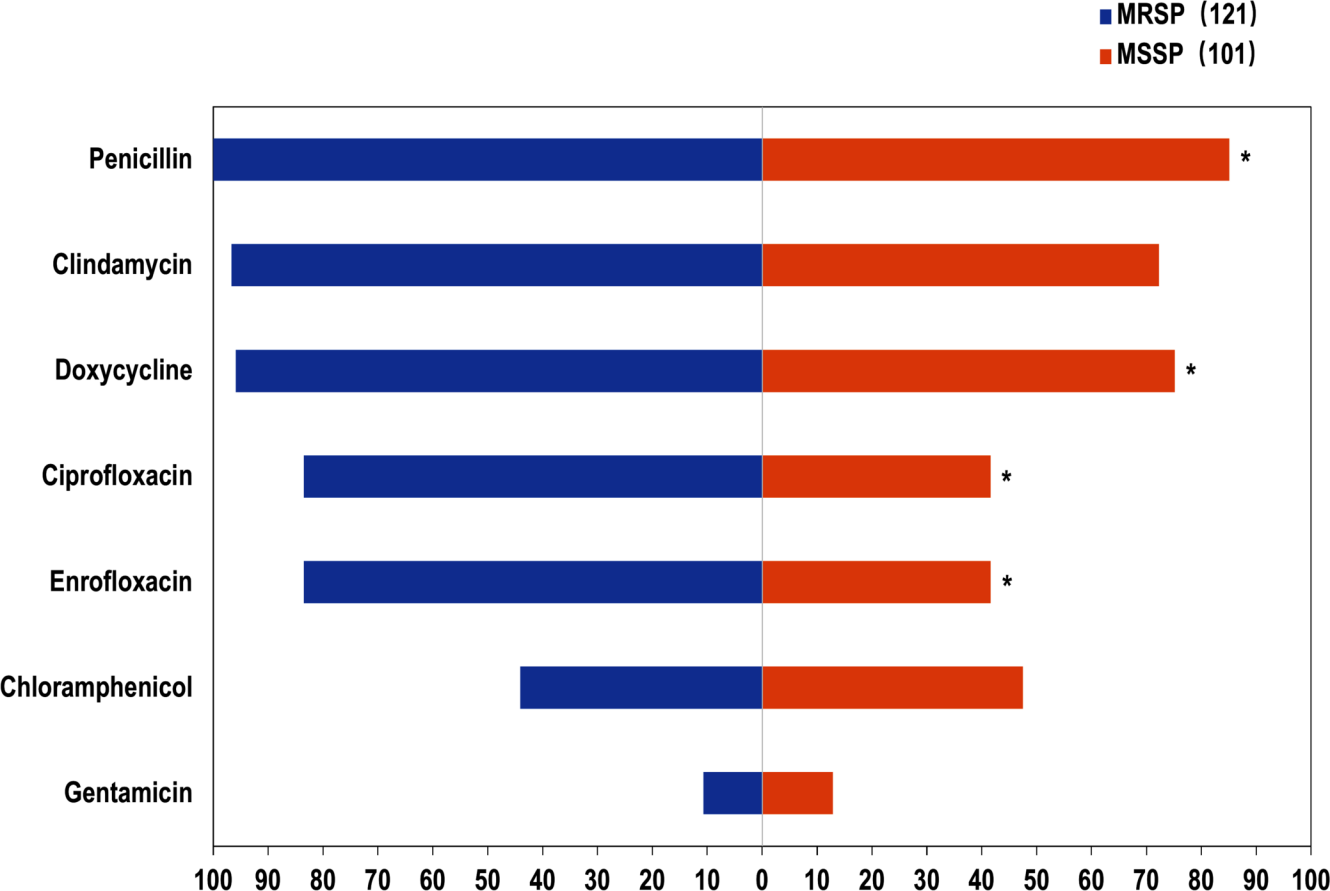
Antimicrobial resistance of MRSP in dogs. *Statistically significant difference (*P* < 0.05) between MRSP and MSSP. MRSP, methicillin-resistant *Staphylococcus pseudintermedius*; MSSP, methicillin-susceptible *Staphylococcus pseudintermedius*.

Methicillin-resistant *S. pseudintermedius* (MRSP) infections are an important cause of morbidity and mortality in pets, posing health risks not only to pet owners but also to employees in the pet industry (45). MRSP has emerged as a multidrug-resistant bacterium, presenting a serious threat in the veterinary field (45, 48). Studies on AMR of *S. pseudintermedius* from dogs and cats in China have primarily focused on South (49) and North China (50). The prevalence of MRSP among all clinical isolates was reported to be 9.9% (89/898) in South China and 3.1% (155/4,973) in North China, with the primary sources of detection being skin and ear swabs (45, 49). MRSP and methicillin-susceptible *S. pseudintermedius* (MSSP) differed in their AMR patterns. The resistance rates of canine MRSP to penicillin, ciprofloxacin, enrofloxacin, and doxycycline were significantly higher than those of canine MSSP (Fig. 6) (45). In addition, the multidrug resistance (MDR) rate of MRSP from dogs was significantly higher than that of MSSP.

Similar to methicillin-resistant *S. aureus* (MRSA), the *mecA* gene in MRSP is located within the staphylococcal chromosomal cassette *mec* (SCC*mec*). In studies of *S. pseudintermedius* isolated from pets in China, the detection rate of SCC*mec* was found to be over 40.0% (45, 49, 50). The detection rate of the aminoglycoside resistance gene *aac(6′)-aph(2*′) ranged from 76.8% (53/69) (49) to 100.0% (33/33) (50). The presence of other resistance genes was also significant, with detection rates exceeding 60.0%. These included the tetracycline resistance gene *tet*(M), the macrolide-lincosamide-streptogramin B (MLS_B_) resistance gene *erm*(B), and the β-lactamase gene *bla*Z. Many of these antimicrobial agents are commonly used in veterinary practice. However, no *Staphylococcus* isolates carrying the multiresistance gene *cfr* have been detected (45, 49, 50). Nevertheless, it is crucial for veterinarians to exercise caution and prudence in the use of these antimicrobial agents.

### *Enterococcus* spp.

**Fig 7:**
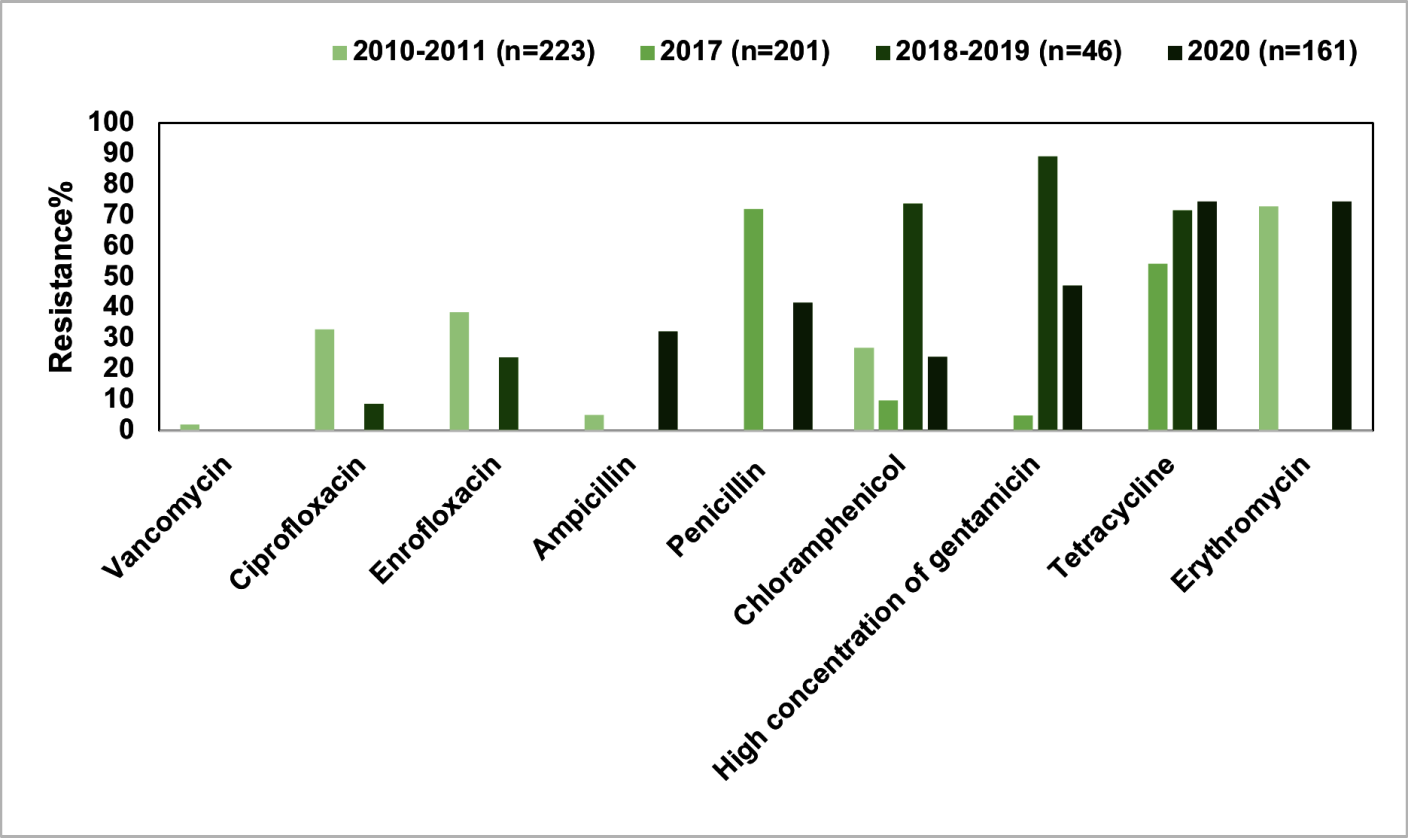
Overall antimicrobial resistance of pet-derived *Enterococcus* spp. in China, 2015–2020.

*Enterococcus* spp. are gram-positive cocci commonly found in natural environments, including livestock farms, and in the intestines of humans and animals. Compared to other gram-positive bacteria, *Enterococcus* spp. showed a broader spectrum of intrinsic resistance (51). This not only complicates veterinary clinical treatments but also poses a significant threat to public health. Studies on pet-derived *Enterococcus* spp. in China mainly involved isolates from fecal samples, with AMR primarily reported in East Central, North, and Northeast China. Over 50.0% of *Enterococcus* spp. exhibited resistance to aminoglycosides, tetracyclines, and macrolides (Fig. 7). The detection rates of the tetracycline resistance genes *tet*(M) and *tet*(L), the MLS_B_ resistance genes *erm*(B) and *erm*(T), and the aminoglycoside resistance genes *aac(6′)-aph(2*′), *aac(6′)-li*, and *ant (6)-la* were also high (>60.0%) (52, 53).

The detection rate of vancomycin-resistant *Enterococcus* (VRE) in pets in China remained low. In 2011, the resistance rate of *E. faecalis* to vancomycin was 2.1% (5/223) in East China (54), which was higher than the detection rate of human-derived VRE. The China Antimicrobial Surveillance Network reported in 2021 that the resistance rates to vancomycin of human-derived *E. faecalis* and *E. faecium* were 0.1% (12/11,215) and 1.4% (186/13,041), respectively (55). In addition, three strains of *E. faecalis* carrying the pRI1 plasmid were found in South China (52). It has been confirmed that this plasmid can co-transfer with conjugative plasmids mediating resistance to vancomycin and streptogramins, thereby facilitating the acquisition and spread of vancomycin resistance among bacteria (56). One of the three *E. faecalis* strains also carried a *vanA*-positive pRUM plasmid (57).

### Other bacterial pathogens

#### Salmonella spp.

*Salmonella* spp. are important human and animal pathogens worldwide, responsible for foodborne infections and zoonotic diseases such as gastroenteritis, systemic infections, and typhoid fever. Companion animals can act as reservoirs for *Salmonella* spp., posing a zoonotic risk to humans (58, 59). In China, studies on pet-derived *Salmonella* spp. mainly focused on fecal isolates, with AMR primarily reported in Northeast, Southwest, and Northwest China. The isolation rate of *Salmonella* spp. from pets in Northwest China ranged from 32.2% (99/307) to 46.7% (36/77), which was higher than the rates reported in other regions, ranging from 2.7% (17/748) to 10.27% (38/370). The AMR of pet-derived *Salmonella* spp. in China showed an initial increase followed by a decline, with 2015 marking a turning point. However, after 2015, resistance rates to ampicillin, tetracycline, doxycycline, sulfamethoxazole/trimethoprim, and chloramphenicol remained above 40% (Fig. S1).

The most common β-lactam resistance genes in *Salmonella* spp. were *bla*_CTX-M_ (65.8%, 25/38), *bla*_TEM_ (68.3%, 28/41 to 97.0%, 35/36), *bla*_PSE_ (47.1%, 8/17), and *bla*_OXA_ (43.9%, 18/41 to 97.0%, 35/36) (60–65). In Southwest China, 48.6% (20/41) of β-lactam resistance gene-positive isolates also carried PMQR genes, with *bla*_TEM_ + *qnrS* being the dominant combination (*n* = 3) (62). Among fluoroquinolone-resistant isolates, *qnrA*, *qnrD*, *qnrS*, and *aac(6′)-Ib-cr* were the most frequently detected genes, with their prevalence exceeding 60.0% in most studies (61, 63, 64). In addition, studies on resistance to other commonly used antimicrobial agents, such as tetracyclines and sulfamethoxazole/trimethoprim, revealed high carriage rates of *tet*(A) (55.3%, 21/38), *tet*(B) (13.2%, 5/38 to 97.0%, 35/36), *tet*(M) (60.5%, 23/38), *sul1* (73.7%, 28/38), *sul2* (65.8%, 28/38), and *sul3* (63.2%, 24/38) in pet-derived *Salmonella* spp. in China (61, 63–65).

#### P. aeruginosa

*P. aeruginosa* is a rod-shaped, gram-negative, glucose-nonfermenting aerobic bacterium. In animals, particularly dogs, it is recognized as a significant pathogen causing infections, such as otitis externa, chronic deep pyoderma, and wound or UTIs (66). In China, *P. aeruginosa* isolates from diseased pets were primarily collected from skin and mucosa infections (28.6%, 22/77), external auditory meatus (20.8%, 16/77), throat (10.2%, 8/77), and wounds (9.1%, 7/77) (67, 68). The overall resistance of pet-derived *P. aeruginosa* in China to antimicrobial agents remained below 25.0%, with resistant isolates primarily reported in South and North China. However, resistance to critical antimicrobial agents exceeded 10.0%. For instance, the resistance rate to colistin was 17.1% (13/76), and the resistance rate to imipenem was 13.5% (8/62) (Fig. S2). A study in North China reported that 14.8% (4/27) of pet-derived *P. aeruginosa* isolates carried class 1 integrons, with all these strains being resistant to 6–10 antimicrobial agents. These integrons harbored variable gene cassettes, including an 1,816 bp region with *aadA4* and *aadA1*, a 2,025 bp region with *bla*_OXA-31_ and *aadA2*, a 2,344 bp region with *aadA1*, *arr-3*, and *catB3*, a 2,570 bp region with *cmlA5*, *cmlA*, and *aadA1*, and an 1,822 bp region with *aac4* and *aadA1* (69). Another study in North China revealed a high level of similarity between the genetic environment of *bla*_IMP-45_ in canine *P. aeruginosa* and an integron found in human ST308 *P. aeruginosa*, suggesting the possibility of transfer of *bla*_IMP-45_-carrying isolates between humans and dogs (69).

#### P. mirabilis

*P. mirabilis*, a member of the Enterobacterales, is a zoonotic pathogen mostly causing secondary infections in humans and animals. As an opportunistic pathogen, it can cause skin infections, respiratory tract infections, UTIs, and gastrointestinal tract infections (70). In China, *P. mirabilis* isolates from diseased pets were primarily collected from the eyes, the nose, and feces. The isolation rate of *P. mirabilis* from pets in China was 28.2% (11/39). Furthermore, these isolates exhibited resistance to gentamicin, chloramphenicol, ciprofloxacin, ampicillin, cefotaxime, ceftriaxone, and sulfamethoxazole/trimethoprim, with resistance rates ranging from 36.4% to 63.6% (Fig. S3).

## CONTAMINATION AND SPREAD OF ANTIBIOTIC-RESISTANT BACTERIA IN THE ENVIRONMENT IN CHINA

### The interspecies transmission between humans and pets

In China, as in other parts of the world, there is a risk of mutual transmission of antimicrobial-resistant bacteria or AMR genes between humans and pets. Studies have demonstrated the potential for interspecies transmission, defined as the co-carriage of commensal isolates in humans and dogs sharing the same environment (71).

Most *E. coli* strains are commensals; however, certain strains can cause life-threatening intestinal and extraintestinal infections in both humans and animals. Importantly, *E. coli* acts as a reservoir for AMR genes, which may spread between humans and animals through close contact, posing a significant public health threat. In 2021, a study in North China identified a dog and its owner carrying identical *bla*_CTX-M_-positive *E. coli* strains, demonstrating the potential for bidirectional transmission (26). Similarly, in 2016, researchers in South China identified five *mcr-1*-positive *E. coli* isolates (ST354) from four dogs and one worker at a pet shop, suggesting the transmission of mcr-1-harboring *E. coli* in shared environments (72). In Romania, a high prevalence of fecal extended-spectrum cephalosporin-resistant *E. coli* was reported in humans and dogs from households and shelters. Notably, two households had dogs and owners carrying *E. coli* isolates with identical Fourier transform infrared spectroscopy spectra, phylogroup, resistance genes, and Inc plasmids, including major lineages such as ST127, ST10, ST155, and ST88 (71).

Similarly, *K. pneumoniae,* another significant resistant pathogen, can asymptomatically colonize the skin, mouth, nasopharynx, and gastrointestinal (GI) tract, with GI colonization serving as a major reservoir for infections (73). The potential for the spread of antimicrobial-resistant *K. pneumoniae* between humans and pets warranted serious attention (74). Although no studies in China have explicitly demonstrated the transmission of *K. pneumoniae* between humans and pets, a study in Lisbon reported that two colonized dogs from the same household shared strains (ST252 and ST1241) with a human cohabitant (75).

Although several studies have provided evidence for the transmission of antimicrobial-resistant bacteria between humans and pets, a clear transmission chain has not yet been established. Given the large number of pets globally, the risk of transmission between humans and pets should not be underestimated. The modes of transmission of antimicrobial-resistant bacteria and AMR genes between pets and humans are complex and require further investigation.

### The risk of spreading antimicrobial-resistant bacteria and AMR genes in shared environments

Beyond transmission between pets and humans, there is also a risk of spreading antimicrobial-resistant bacteria and AMR genes in shared environments, such as homes and pet hospitals. These environments, where pets and humans frequently interact, and where sick animals are often in contact with the surroundings, are critical areas for monitoring. This risk is supported by multiple studies investigating antimicrobial-resistant bacteria in various settings.

In Southwest China, *E. coli* and *Enterococcus* spp. were isolated from 26.5% (66/249) to 70.5% (91/129) and 59.8% (149/249) to 79.8% (103/129) of environmental samples, respectively, collected from pet hospitals, pet clinics, and pet trading markets. The highest isolation rates for both bacteria were observed in animal cages (*E. coli*: 65.9%, 62/94; *Enterococcus* spp.: 80.8%, 72/94) (76). Similar AMR patterns were noted in other pathogens, such as *Acinetobacter* spp., which are known to cause severe infections, including skin and soft tissue infections, UTIs, and secondary meningitis (77). In South China, a study investigating environmental samples from five pet hospitals found *Acinetobacter* spp. in 1.9% (14/730) of samples, primarily from operating rooms (*n* = 4), immunization rooms (*n* = 4), and dog cages (*n* = 6). Notably, one multidrug-resistant *A. pittii* strain was identified, carrying carbapenem resistance genes, including *bla*_NDM-1_, *bla*_OXA-500_, and *bla*_OXA-727_ (78).

Similarly, a study conducted in the United Kingdom, which collected environmental and clinical fecal samples from two veterinary hospitals (equine and small animal), identified EKAPE gram-negative pathogens (*Enterococcus faecium*, *K. pneumoniae*, *Acinetobacter baumannii*, *P. aeruginosa*, and *Enterobacter* spp.) as the most prevalent extended-spectrum cephalosporin-resistant isolates. These included *Enterobacter cloacae* complex (21.7%), *P. aeruginosa* (20%), *K. pneumoniae* (15.9%), and *A. baumannii* complex (13.6%), followed by *E. coli* (12.2%). Many isolates harbored beta-lactamase- and ESBL-encoding genes, with mobile carbapenem resistance genes detected in three environmental *Acinetobacter* spp. isolates carrying *bla*_OXA-23_ and one clinical *E. coli* isolate with *bla*_OXA-48_, highlighting the potential for environmental dissemination of resistance to the last-resort antimicrobial agents (79).

*Staphylococcus* spp., another significant zoonotic pathogen, often survive well in veterinary environments. In South China, *S. aureus* was most frequently detected in air dust (19.1%, 13/68), followed by medical device surfaces (10.8%, 4/37) and environmental surfaces (4.3%, 6/138) in a pet hospital (80). Similarly, in Germany, MRSA and MRSP isolates were identified among employees and small animal hospital environments. MRSA was found in five nasal swabs (5.2%, 5/96) from 55 employees and in six environmental samples (8.2%, 6/73), while MRSP was detected in nasal and hand swabs of two employees (2.1%, 2/96) and in three environmental samples (4.1%, 3/73). All environmental isolates were found in high-traffic areas, such as dog wards, waiting rooms, and triage rooms (81).

A related study in Switzerland examined environmental contamination with multidrug-resistant organisms (MDROs) and MDRO carriage among personnel in seven companion animal clinics. Carbapenemase-producing (CP) *Enterobacterales* were detected in two clinics, one of which exhibited extensive contamination with CP *K. pneumoniae* (ST11, *bla*_OXA-48_) and MRSP (ST551, *mecA*), particularly in intensive care units, consultation rooms, and on utensils. Two employees were found to carry CP *E. coli* closely related to environmental isolates (ST410, *bla*_OXA-181_) and patient-derived isolates (ST167, *bla*_NDM-5_). In addition, MRSA (ST225, *mecA*) and MRSP (ST551, *mecA*) with identical sequence types and similar resistance profiles were identified in both employees and the environment of the two clinics (82).

These findings underscore the significant public health risks posed by AMR transmission in shared environments, including homes and veterinary hospitals. The detection of transmissible carbapenem resistance in pathogens, such as *Acinetobacter* spp. and *E. coli* highlights the urgent need for enhanced monitoring and intervention. Improving household hygiene and implementing stringent disinfection protocols in pet hospitals are essential steps to mitigate these risks and reduce the dissemination of AMR pathogens across human and animal populations.

## PROSPECT

The rise of antimicrobial-resistant bacteria in pets from China poses a growing public health concern due to the zoonotic potential and resistance to commonly used antimicrobials. Key pathogens, including *E. coli*, *K. pneumoniae*, *Staphylococcus* spp., *Enterococcus* spp., *Salmonella* spp., *P. aeruginosa*, and *P. mirabilis*, have shown varying levels of resistance. *E. coli* exhibited high resistance to β-lactams and fluoroquinolones, with ESBL production exceeding 40.0% in some regions, often linked to *bla*_CTX-M_ genes. *K. pneumoniae* showed significant resistance to aminoglycosides and cephalosporins, while carbapenem and colistin resistance, though rare, were associated with transferable genes like *bla*_NDM-5_ and *mcr-1*. MRSP displayed MDR, with resistance to fluoroquinolones and tetracyclines exceeding 50.0%. *Enterococcus* spp. showed high resistance to tetracyclines, macrolides, and aminoglycosides, with the detection of *tet*(M), *erm*(B), and *aac(6′)-aph(2'*) genes. Although VRE detection rates remained low, plasmids carrying the *vanA* gene cluster were identified, highlighting the potential for horizontal gene transfer and the spread of vancomycin resistance. *Salmonella* spp. showed high resistance to tetracyclines and sulfamethoxazole/trimethoprim, frequently carrying β-lactam and quinolone resistance genes. *P. aeruginosa* maintained resistance below 25.0% for most drugs but showed notable resistance to colistin and imipenem, often linked to class 1 integrons. *P. mirabilis* exhibited resistance to aminoglycosides, β-lactams, and fluoroquinolones. These findings highlight the need for improved AMR surveillance and stricter antimicrobial use in veterinary practices to reduce the risk of zoonotic transmission.

The reviewed studies highlight the critical role of shared environments, such as homes and veterinary hospitals, in the transmission of antimicrobial-resistant bacteria and AMR genes between humans and pets. Pathogens like *E. coli*, *K. pneumoniae*, *Acinetobacter* spp., *Enterococcus* spp. and *Staphylococcus* spp. frequently harbor resistance genes, including those conferring resistance to last-resort antimicrobial agents like carbapenems and colistin. These pathogens pose a significant zoonotic risk, particularly in households with high levels of pet-human interaction or among immunocompromised individuals, such as the elderly, children, or patients with underlying health conditions. In such populations, infections caused by resistant pathogens can lead to severe clinical outcomes, including prolonged illness, limited treatment options, and increased mortality rates. The detection of these pathogens in high-traffic areas and across both environmental and clinical samples underscores the potential for cross-species and environmental dissemination. Shared environments, including homes, veterinary hospitals, and public spaces, such as pet trading markets, act as reservoirs and transmission hubs for resistant bacteria. These settings provide opportunities for direct transmission between pets and humans, as well as indirect transmission through contaminated surfaces, air, and shared equipment. For instance, pet bedding, feeding bowls, and toys may serve as fomite carriers of resistant bacteria, amplifying their spread within households. This evidence underscores the need for strengthened hygiene measures, routine AMR monitoring, and robust disinfection protocols in shared human-animal environments to mitigate the spread of AMR pathogens and safeguard public health.

Despite the progress made, the monitoring of AMR in pet-derived bacteria in China started relatively late and still faces several key challenges. First, because of China’s vast geographical diversity, most current research on pet-derived antimicrobial-resistant bacteria is regionally or city-focused, making it challenging to establish, coordinate, and organize a comprehensive national surveillance network. Second, research has primarily concentrated on specific infection sample types or bacterial species, lacking continuous and systematic monitoring of AMR across the full spectrum of bacterial pathogens in pets. Third, different bacterial species require different antimicrobial agents for treatment, resulting in gaps in monitoring data for important antimicrobial agents.

To address these challenges, the establishment of a national AMR network, CARPet, was of particular importance. CARPet aims to ensure comprehensive coverage by selecting representative cities from different regions of China and incorporating a wide variety of clinical sample sources and bacterial species and genera, with a particular focus on important zoonotic pathogens. It is essential for CARPet to monitor not only the commonly used antimicrobial agents in veterinary practice but also critically important antimicrobials in human medicine, such as carbapenems, tigecycline, and vancomycin, to effectively track and mitigate the spread of AMR.

Moreover, accurate diagnosis of AMR in veterinary medicine is critical for effective management and treatment. Traditional methods, such as bacterial culture, antimicrobial susceptibility testing, and whole-genome sequencing, remain essential but are often time-consuming and may not deliver rapid results. Emerging technologies—such as MALDI-ToF mass spectrometry, machine learning, metagenomic sequencing, and metatranscriptomic sequencing—offer new opportunities for more rapid AMR diagnostics. However, before these technologies can be widely adopted in veterinary practice, rigorous benchmarking is needed to ensure their reliability and effectiveness.

In conclusion, while significant progress is being made, advancing national surveillance networks and adopting innovative diagnostic technologies are essential steps to strengthen AMR monitoring and management in veterinary medicine. These efforts ensure a more efficient and accurate approach to combating this global threat.

## SUPPLEMENTAL MATERIAL

Supplemental material aac.01657-24-s0001.docx Text S1 and S2; Figures S1 to S3.

Table S1 aac.01657-24-s0002.xlsx Antimicrobial resistance data and associated information for pet-derived bacteria in China (2000-2020).

ASM does not own the copyrights to Supplemental Material that may be linked to, or accessed through, an article. The authors have granted ASM a non-exclusive, world-wide license to publish the Supplemental Material files. Please contact the corresponding author directly for reuse.
