# Supplementary material for: Antimicrobial resistance of pet-derived bacteria in China, 2000–2020
Source: Antimicrob Agents Chemother. 2025 Mar 26;69(5):e01657-24. doi: 10.1128/aac.01657-24 (PMC12057338; doi:10.1128/aac.01657-24)
Supplement: Supplemental material — Text S1 and S2; Figures S1 to S3. [file aac.01657-24-s0001.docx]

Supplementary Text

S1. Literature Review and Data Harmonization

We searched for veterinary literature reporting antimicrobial resistance (AMR) rates in China on two international databases (PubMed and ISI Web of Science), as well as the leading Chinese-language academic search engine – China’s National Knowledge Infrastructure (CNKI). We focused on four common indicator pathogens: *Escherichia coli*, *Klebsiella pneumoniae*, *Staphylococcus* spp., *Enterococcus* spp., *Salmonella* spp., *Pseudomonas aeruginosa* and *Proteus mirabilis*. The search was conducted on November 20, 2020, and included all studies published between 2000 and 2020. The search query used the generic structure: (Resistance) AND (Bacterial Species) AND (Animals and Sample types). The key words used on CNKI was as follows, with all possible combinations of drugs, pathogens, and animals: (‘抗生素’ + ‘抗菌’ + ‘兽药’ + ‘兽用药’ + ‘兽用抗生素’ + ‘用药’ + ‘抗微生物’) AND TI = (*Escherichia* + (*E. coli*) + coliform + pneumoniae + (*K.* *pneumoniae*) *+* *Enterococcus* + enterococci + VRE + (*E. faecalis*) + (*E. faecium*) + (*S. aureus*) + *Staphylococcus* + MRSA + MSSA+ *Salmonella* spp. + *Pseudomonas aeruginosa + Proteus mirabilis*+ ‘埃希菌’ + ‘大肠杆菌’ + ‘大肠菌’ + ‘克雷伯氏菌’ + ‘肠球菌’ + ‘葡萄球菌’ + ‘沙门菌’ + ‘铜绿假单胞菌’ + ‘奇异变形杆菌’) AND TI= (‘动物’ + ‘犬’ + ‘猫’ + ‘宠物’ + ‘伴侣动物’). The full search query used in the advanced search functionality was: (TI = key words) OR (KY = key words) OR (AB = key words), where TI, KY, and AB stand for title, keywords and abstract, respectively.

The key words used on PubMed and ISI Web of Science was as follows: (antimicrobial resistance OR resistance OR susceptibility OR antibiogram OR antibiotic susceptibility testing OR antibiotic OR antimicrobial OR antibacterial) AND (escherichia OR E. coli OR pneumoniae spp. OR K. pneumoniae OR enterococcus OR enterococcus spp. OR enterococci OR VRE OR E. faecalis OR E. faecium OR S. aureus OR staphylococcus OR Staphylococcus spp. OR MRSA OR MSSA OR salmonella OR Salmonella spp. OR Pseudomonas OR Pseudomonas aeruginosa OR Proteus OR Proteus mirabilis) AND (animal OR pet OR dog OR cat OR companion animal) AND (China). On PubMed, the key words were directly used as the full search query. On ISI Web of Science, the full search query was specified as TITLE-ABS-KEY = (key words), TITLE-ABS-KEY stands for title, abstract and key words.

From PubMed, ISI Web of Science and CNKI, the literature search resulted in 6,457 documents from China. Abstracts were screened manually. We removed reviews, meta-analysis studies, and publications that did not report resistance rates, and retained 1,875 publications as potentially relevant point prevalence surveys (PPS) to read in full. Among these, we excluded: surveys in which resistance rates were pooled between pathogens, and studies without information on sampling locations. We further excluded those PPS with small sample sizes, extremely high or low antimicrobial resistance rates or obvious errors in antimicrobial resistance data. Finally, 528 antimicrobial resistance rates were extracted from 38 PPS for drug-pathogens combinations recommended for susceptibility testing by the WHO AGISAR consortium 1.

S2. Trends in AMR

We calculated the weighted arithmetic mean of AMR to summarize trends in resistance across multiple antimicrobial agents and bacterial species. For each bacterium, we assessed the significance of the temporal trends of antimicrobial resistance rates between 2000 to 2020 using a weighted arithmetic mean model, weighted by the number of bacteria in each survey accounted for the proportion of total number of bacteria.

*‾R* : weighted arithmetic mean of AMR for each bacterium

*R_n_*: the resistance rate of each bacterium–drug (antimicrobial class) combination

*n_n_*: the number of bacteria in each survey


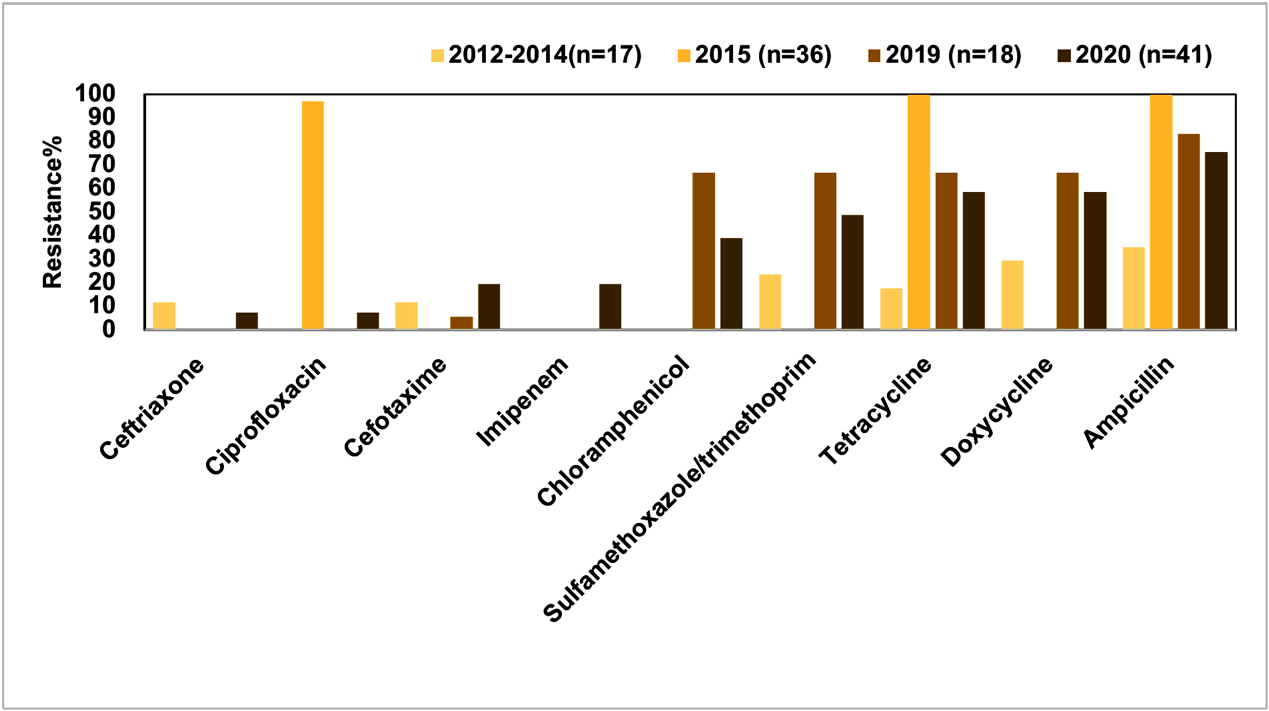


Figure S1. Overall antimicrobial resistance of pet-derived *Salmonella* spp. in China, 2012-2020.


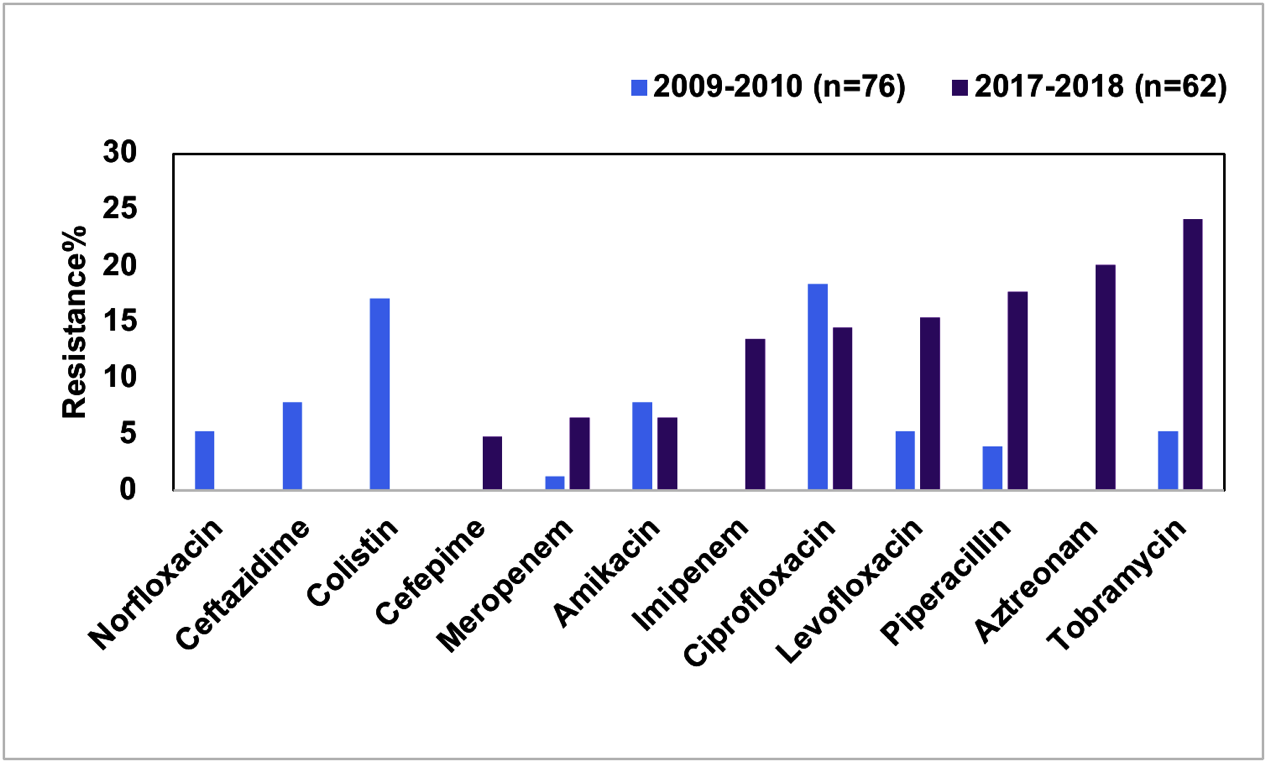


Figure S2. Overall antimicrobial resistance of pet-derived *Pseudomonas aeruginosa* in China, 2009-2018.


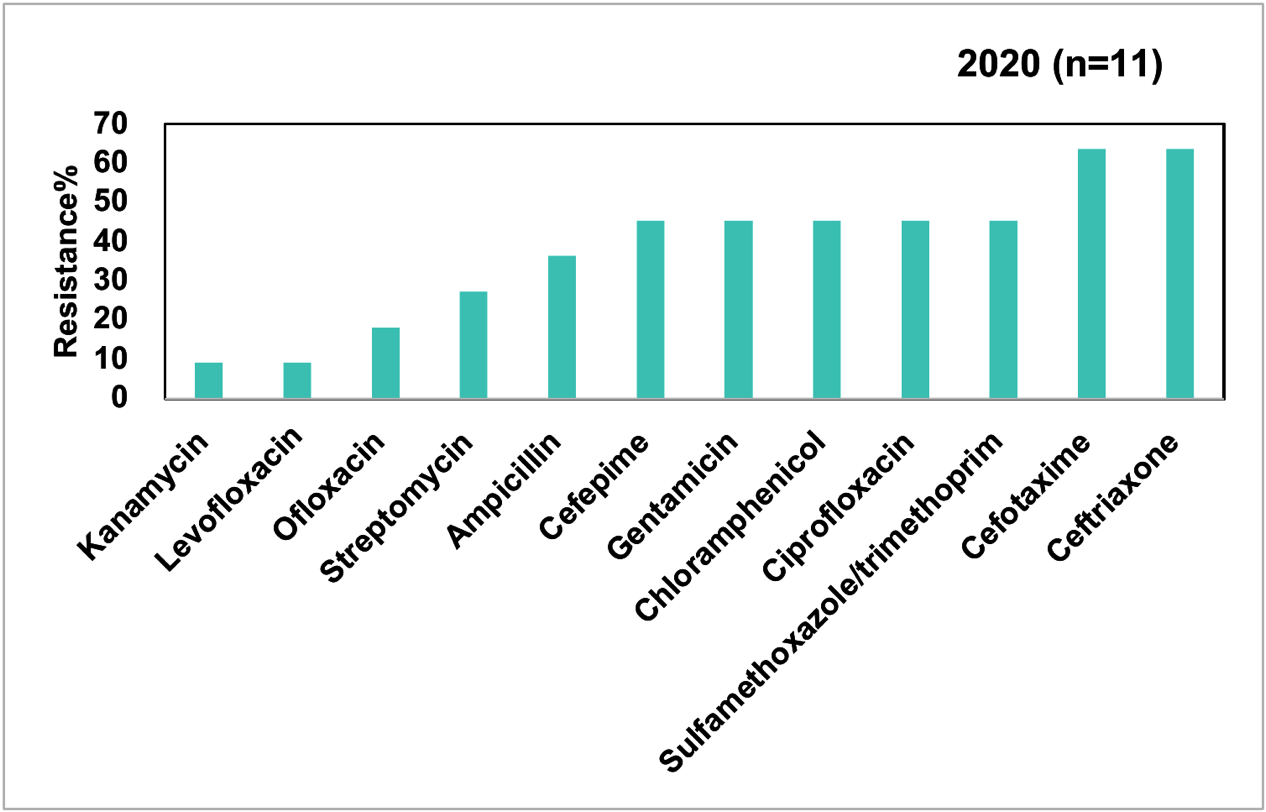


Figure S3. Overall antimicrobial resistance of pet-derived *Proteus mirabilis* in China, 2020.
